# CAR-T therapy: Trailblazing CAR(ing) in cancer treatment

**DOI:** 10.18632/oncotarget.28836

**Published:** 2026-02-20

**Authors:** Uzma Saqib, Monika Pandey, Krishnan Hajela

**Keywords:** CAR-T therapy, cancer, therapeutic approaches

Recent strategies to combat cancer appear to offer a silver lining amidst the prevailing challenges [1]. Given the small percentage of patients cured from advanced-stage disease, there is still a considerable journey ahead. Nonetheless, novel therapeutic approaches have, in some cases, demonstrated remarkable success in improving patient survival and longevity [2].

Among these, CAR T-cell therapy has emerged as a particularly promising cancer-specific treatment strategy [3]. This approach involves genetically modifying a patient’s T cells in the laboratory so they can recognize and destroy cancer cells, even in hard-to-treat malignancies. The concept can be explained through a simple analogy: cancer cells are likened to an invading enemy at the borders of a country. Normally, the body’s T cells act as soldiers, but they may not be sufficiently equipped to counteract the enemy’s advanced weaponry (antigens). To overcome this, oncologists recall these soldiers (T cells) and arm them with specialized weapons called chimeric antigen receptors (CARs), that target the enemy’s artillery. Once multiplied in large numbers, these enhanced soldiers are redeployed to the battlefield (the patient’s body), where they attack and eliminate the cancer cells with improved precision.

**Figure 1:**
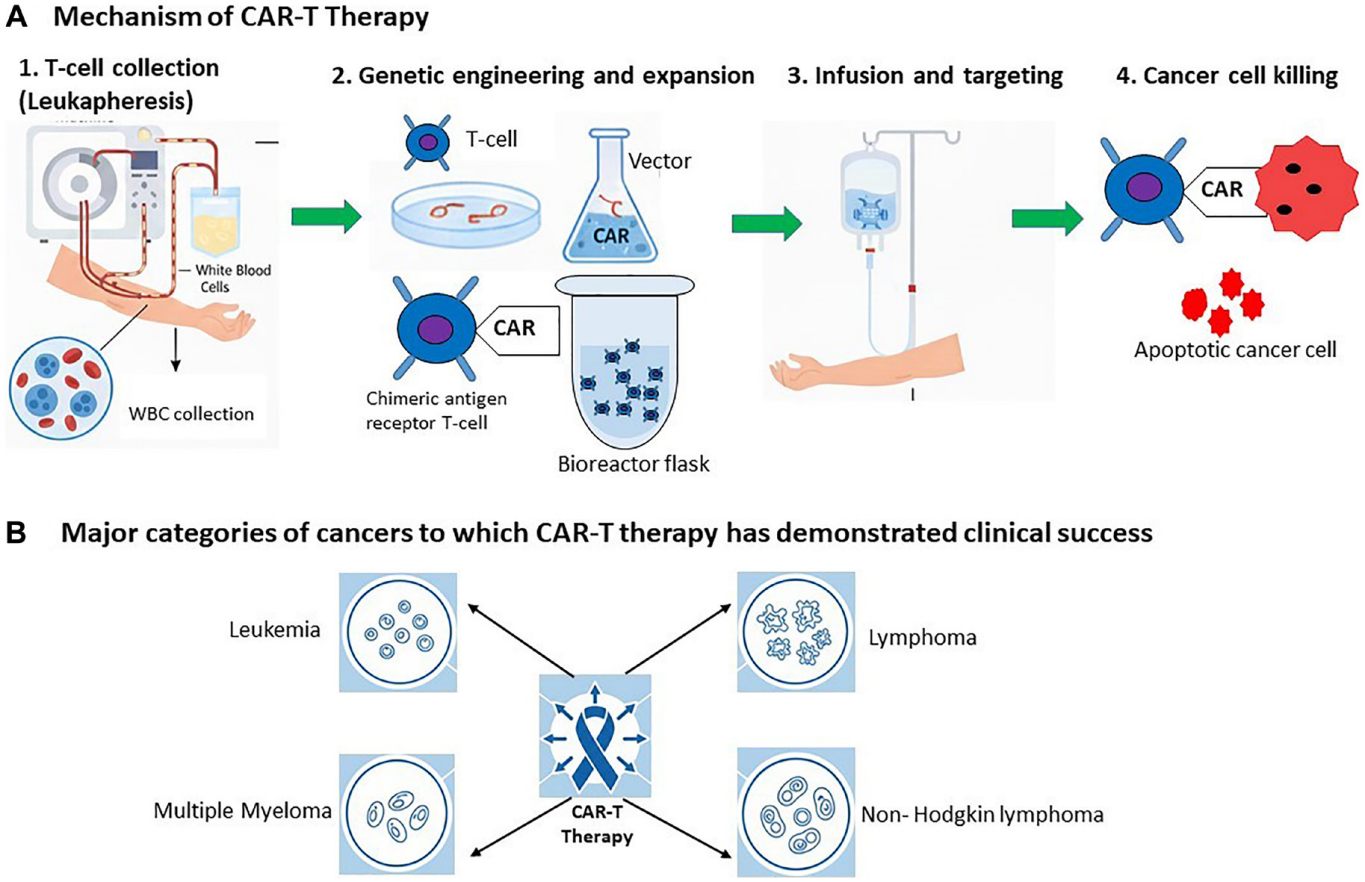
Overview of CAR T-cell therapy workflow and clinical applications. (**A**) The CAR-T design comprises three major steps: T-cell collection via leukapheresis; genetic modification and ex vivo expansion of T cells engineered to express a CAR; and subsequent reinfusion following lymphodepleting chemotherapy. After administration, CAR-modified T cells recognize tumor-associated antigens and initiate targeted cytotoxicity. (**B**) Clinical implementation has demonstrated significant efficacy across hematologic malignancies, including leukemia, lymphoma, multiple myeloma, and relapsed or refractory non-Hodgkin lymphoma.

Clinically, CAR T-cell therapy involves three main stages [4]: (i) T cell collection, in which white blood cells are extracted from the patient through leukapheresis; (ii) editing and expansion, where the collected T cells are genetically engineered with CAR genes and expanded in culture over several weeks; and (iii) infusion, where the modified cells are reintroduced into the patient, typically following chemotherapy to reduce competing immune cells. Once infused, the CAR T cells bind to their target antigens and initiate cancer cell destruction (Figure 1A).

CAR T-cell therapy has shown success across various malignancies (Figure 1B), including leukemia [5], lymphoma [6], and multiple myeloma [7] etc. Recent clinical trials have even demonstrated superior outcomes compared to standard treatment in patients with non-Hodgkin lymphoma [8], positioning CAR T-cell therapy as a potential replacement for chemotherapy as the second-line standard of care. Not only haematological malignancies, but solid-tumor applications of CAR-T therapy have also shown encouraging outcomes. Recent phase I reports have documented measurable clinical responses in glioblastoma [9] and breast cancers [10, 11], with additional reviews supporting the growing feasibility of CAR-T strategies in solid tumors [12]. Similarly, a clinical study in thoracic cancers demonstrated that mesothelin-targeted CAR-T cells can achieve promising safety and anti-tumor activity across multiple patient cohorts [13]. Furthermore, a pivotal phase 2 trial evaluating CT041-ST-01 in gastric and gastro-oesophageal junction cancers has shown encouraging signs of efficacy alongside an acceptable safety profile [14].

**Figure 2:**
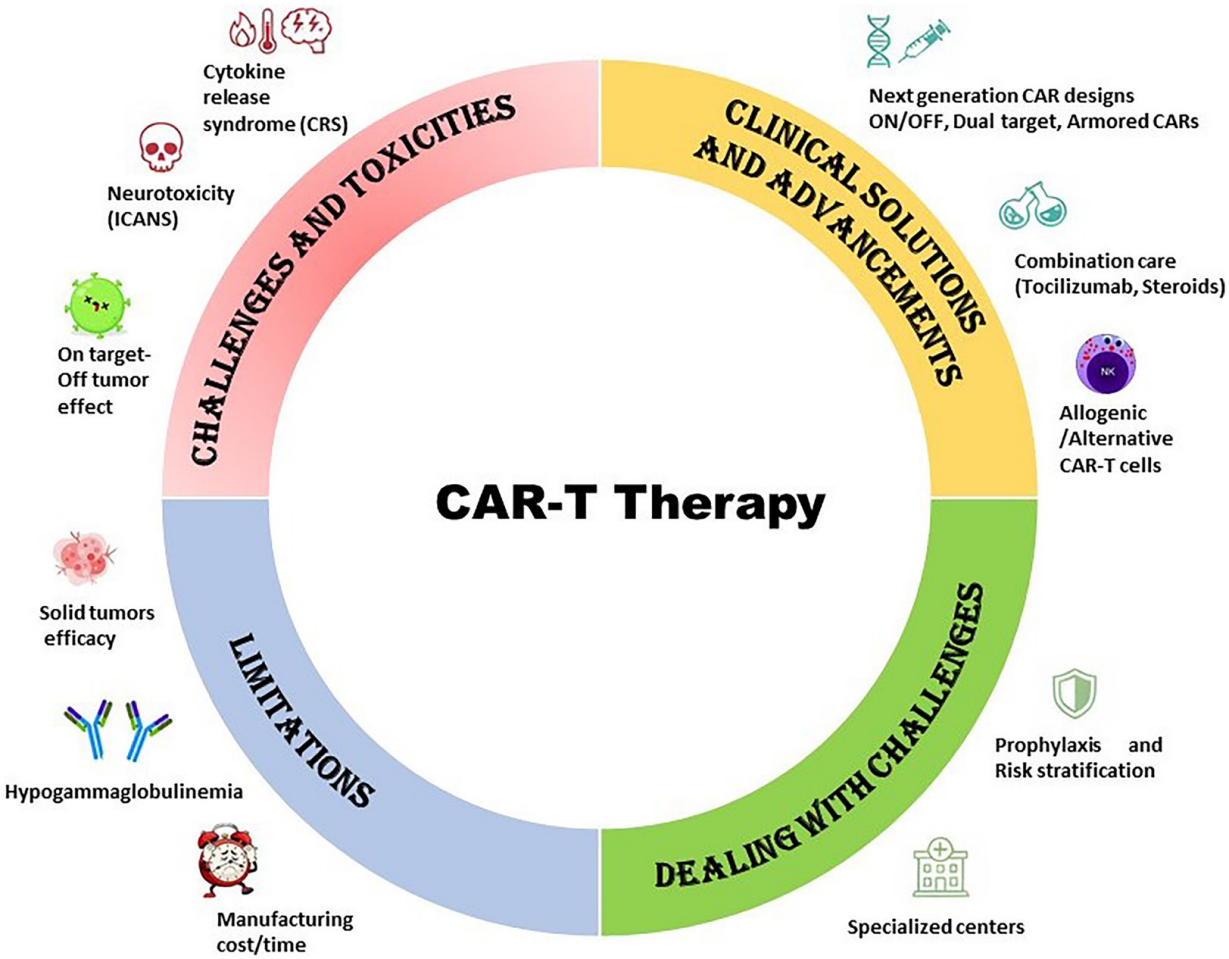
Challenges and emerging advancements in CAR-T therapy. The schematic representation outlines the major limitations associated with CAR-T treatment, including various toxicities, limited efficacy in solid tumors, on-target/off-tumor effects, hypogammaglobulinemia, time constraints and associated costs. The challenges could be substantially addressed with key advancements such as next-generation CAR constructs, optimized supportive care including tocilizumab and corticosteroids, and the development of allogeneic or alternative CAR-T platforms. Enhanced clinical management strategies such as prophylactic measures and the use of specialized treatment centres could further contribute to improving safety, feasibility, and overall therapeutic outcomes.

Despite its promise, CAR T-cell therapy faces several critical challenges (Figure 2). Major clinical concerns include cytokine release syndrome (CRS) and neurotoxicity [15], both of which critically impact patient safety. Additionally, CAR-T resistance in B-cell malignancies [16] and multiple myeloma [17] highlights that only a limited percentage of patients achieve long-term remission. These failures may arise from host- and tumor-intrinsic factors, the surrounding micro and macro milieu, as well as intrinsic limitations of the CAR-T therapy itself [18]. The application of CAR-T therapy in solid tumors remains particularly constrained due to the scarcity of precise tumor-specific antigens, the presence of a profoundly immunosuppressive tumor microenvironment, restricted T-cell trafficking and expansion within tumors, and the potential for severe toxicities [19]. Finally, one of the most formidable barriers is accessibility, as socioeconomic and racial disparities continue to limit the availability of CAR-T therapy, leaving only a small fraction of eligible patients able to receive it [20].

Nevertheless, ongoing research is actively optimizing CAR-T design (Figure 2), delivery, and safety management, progressively overcoming these obstacles and reinforcing its potential to transform cancer care in the coming years.
